# Controlled drug delivery and cell adhesion for bone tissue regeneration by Keplerate polyoxometalate (Mo_132_)/metronidazole/PMMA scaffolds

**DOI:** 10.1038/s41598-022-18622-w

**Published:** 2022-08-24

**Authors:** Hamid Taghiyar, Bahram Yadollahi, Abolghasem Abbasi Kajani

**Affiliations:** 1 Department of Chemistry, University of Isfahan, Isfahan, 81746-73441 Iran; 2 Department of Biotechnology, Faculty of Biological Science and Technology, University of Isfahan, Isfahan, 81746-73441 Iran

**Keywords:** Drug discovery, Molecular medicine, Chemistry

## Abstract

The aim of this study is to fabricate a new scaffold appropriate for tissue regeneration with antimicrobial activity and ability of controlled drug delivery. In this regard, scaffold nanofibers were produced using poly (methyl methacrylate) (PMMA), Mo_132_ as a Keplerate polyoxometalate and metronidazole. The final scaffolds, obtained by electrospinning, represent the intrinsic features including exceptional doubling tensile strength, high hydrophilicity (126 ± 5.2° to 83.9 ± 3.2° for contact angle and 14.18 ± 0.62% to 35.62 ± 0.24% for water uptake), proper bioactivity and cell adhesion. Moreover, the addition of Mo_132_ and metronidazole enhances the biodegradation rate of resulted scaffolds compared to the pure PMMA membrane. The controlled release of metronidazole over 14 days efficiently inhibits the colonization of anaerobic microorganisms. Overall, the results demonstrate high potential of Mo_132_ and metronidazole-loaded PMMA scaffold for guided bone regeneration/guided tissue regeneration.

## Introduction

Guided bone regeneration (GBR)/guided tissue regeneration (GTR) procedures are developing into a standard method for bone or tissue therapy. These procedures use a barrier membrane to direct the growth of new bone or tissue^1,2^. Bone defects are a major health concern due to the damage of bone tissues by bacterial colonization at the wound site. Therefore, a high biocompatible membrane for local delivery of antibiotics is desired^3^. On the other hand, one of the most challenging issues in nano-biotechnological studies is the lack of effective and safe carriers in drugs delivery^4–6^. Many compounds with specific properties have been used for drug delivery so far, notably polyoxometalates (POMs) are also among them^7–9^.

POMs, as polymetallic metal oxides based on early transition metals, are intriguing biomedical agents due to their versatile bioactivity, molecular structure, composition, solubility, electrical properties, and reactivity that endow antibacterial, anticancer, and antiviral functions^10–14^. The ability to synthesize the POMs with tunable molecular structure and physicochemical properties from readily available precursors is the unique advantage of POMs over current drugs^15–18^. Although POMs represent the promising anticancer and antiviral activities, their biomedical application is limited. This is due to their toxic side effects at higher dosages and the unspecific interactions with biomolecules via their negatively charged structures with a rather homogeneous surface of closely packed oxygen atoms^19,20^. Therefore, development of the novel, safe and innovative ways for safer and more effective POM therapy through enhancement of their bioactivity and reduction of their toxic side effects is of great interest^21^. Hence, POMs are the potential candidates for use in the biological sciences, including drug delivery and cell adhesion for bone tissue regeneration.

Additionally, it should be noted that compounds with three-dimensional networks containing nanoscale holes and channels can serve as filters and traps/hosts for molecular guests. These compounds can be used in separation, storage, and transportation of drugs^22,23^. The porous spherical nanocapsules and discrete nanosized species of the type {(M^VI^)M^VI^_5_}_12_(linker)_30_ (M = Mo or W and linker = Mo_2_, Fe, VO, Cr or Ln), that are called “Keplerate”, are structurally well-defined. Keplerate POMs can be considered as artificial cells because they are able to interact specifically with their environment^24–28^. These anionic porous nanocapsules can be obtained with different counter ions (mostly have facile syntheses) with the above-mentioned characters. More importantly, the twenty {Mo_9_O_9_}-type pores with crown ether-like functions can therefore be closed noncovalently in Keplerates by plugging them with cationic guests in a supramolecular fashion^29,30^. The Mo_132_, (NH_4_)_42_[Mo^VI^_72_Mo^V^_60_O_372_(CH_3_COO)_30_(H_2_O)_72_], is a Keplerate with hollow giant isopolyoxomolybdate core that can be covered by hydrophobic or hydrophilic shells of cations via self-assembly^31,32^. A route for embedding negatively charged nanocapsules into lipid bilayer membranes via self-assembly have been demonstrated via molecular dynamics simulations^33^. Moreover, the toxicity of Mo_132_ is studied by analysis of animals’ peripheral blood and its usage as container or core for transport of drugs has been proposed^34^. Thus, it is possible to use the Keplerates in drug delivery by appropriate counter ions (surfactants).

Another Key issue in GBR is choosing the proper antibiotic. Metronidazole (MTN), as a kind of nitro imidazole, has enjoyed considerable attention and could be used for this purpose. MTN has shown a tendency to penetrate and accumulate in regions of tumors. This compound can undergo bioreduction to electrophilic substances, and so, damage proteins and nucleic acids^35,36^. In the previous studies, MTN and its derivatives have exhibited the attractive possibility to act as carriers in targeted drug delivery for cancer therapy^37–40^.

In GBR procedure, the suitable membrane should consist of biodegradable and biocompatible materials with appropriate pore size and porosity, adequate mechanical and physical properties, and osteoconductivity. Using the polymeric micro/nanostructures could be a promising idea that could adequately address these requirements. Polymeric micro/nanostructures have enjoyed considerable attention as drug delivery systems^41,42^. The systems using polymeric micro/nanostructures are based on the increased surface area of drug carriers and controls on the drug-dissolution rates. In this regard, electrospinning could be used for conversion of supramolecular mixtures to different micro/nanostructures with several desirable properties such as high surface to volume ratio, flexible surface functional groups, adjustable surface properties, and excellent mechanical performance. These micro/nanostructure fibers could exhibit appropriate options for different applications. Therefore, in order to use POMs in these polymeric micro/nanostructure drug delivery systems, the synthesis of suitable supramolecular gels is particularly important.

**Figure 1:**
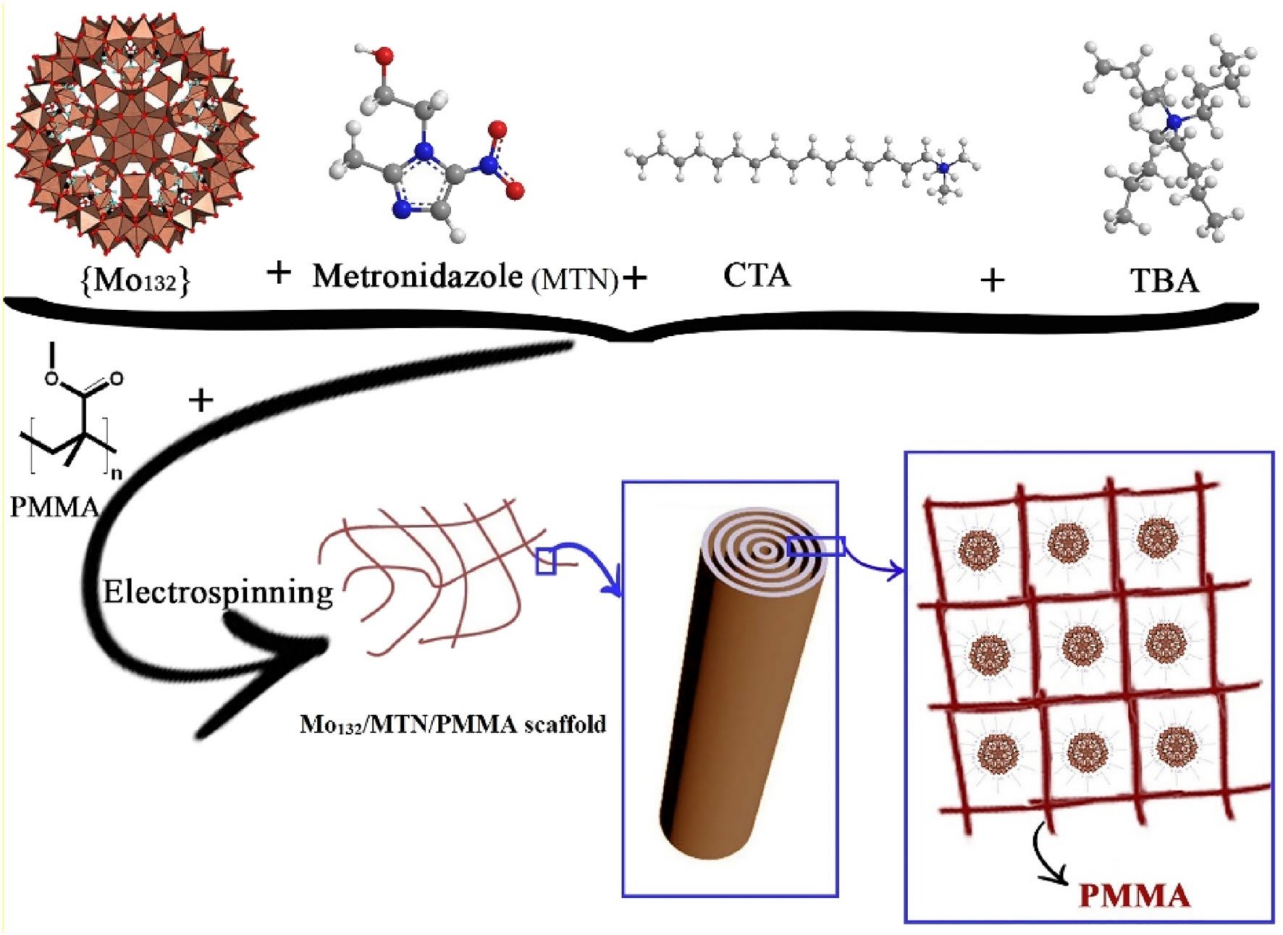
Schematic representation of the used synthesis and fabrication strategy for the development of NF nanofiber scaffolds.

In present study, a supramolecular gel is synthesized by self-assembly of surfactants and MTN encapsulated Keplerate POM (Mo_132_). Afterwards, the partially polymerized gel is converted into micro/nanofibers by electrospinning to provide a platform for controlled delivery of embedded POM and MTN (Fig. 1). The mechanical and physicochemical properties, drug release behavior, and in-vitro biocompatibility of these nanofibers are investigated to evaluate their potential application for GBR membrane.

## Materials and methods

3-(4,5-dimethylthiazol-2-yl)-2,5-diphenyltetrazolium bromide (MTT; Sigma, Saint Louis, USA), hydrazine sulfate, ammonium heptamolybdate tetrahydrate, ammonium acetate, acetic acid, tetrabutylammonium bromide (TBAB), cetyltrimethylammonium bromide (CTAB), methyl methacrylate (MMA), benzoyl peroxide (BPO), and all of the used solvents were purchased from Sigma-Aldrich or some of the other chemical companies. All of the other chemicals were analytical grade and applied without further purification. Metronidazole (MTN) was purchased from Amin pharmacy (Iran).

### Characterization methods

To confirm the chemical composition of nanofibers, Fourier transform infrared spectroscopy (FT-IR; JASCO FT/IR-680 PLUS) was performed over a range of 400–4000 cm^−1^ and resolution of 2 cm^−1^. Powder X-ray diffraction (XRD) was conducted using X'Pert Pro X-ray diffractometer (Phillips, Netherlands) with CuK" radiation (k = 0.15406 nm) at a generator voltage of 40 kV and a current of 40 mA. The thermal stability of nanofibers was investigated by thermogravimetric analysis (TGA; Rheometric scientific 1998, USA). The nanofibers were heated from 30 to 800 °C at a heating rate of 10 °C/min and the weight losses of samples during the test were used for calculations. The surface morphology of nanofibers was observed by scanning electron microscopy (SEM) analysis using a Philips XL30 SEM. The nanofibers were coated with a thin layer of gold before being observed under the microscope and fiber diameter size was measured using the Image J software on SEM micrographs at 20 random locations. The surface areas were calculated using the BET equation and pore size distribution curves were calculated via the BJH method. The pore volume was estimated to be up to P/P_0_ = 0.98.

The mechanical properties of various nanofibers were evaluated by tensile strength tests (INSTRON, Zwick, The United Kingdom) with 10 N load capacity at a rate of 10 mm/min. Samples with dimensions of 70 mm × 10 mm were prepared in rectangular pieces. At least 3 samples were prepared for each nanofiber composition. Tensile strength, tensile modulus, and strain at break were determined from the stress–strain curves. Tensile modulus was determined from the slope of initial linear portion of stress–strain curve, while strain at break was obtained when samples failed. Samples were evaluated to obtain the mean and standard deviation (SD) for each nanofibrous. Electrical conductivity was measured by Schott conductivity meter (CG885). The electrical conductivity of the scaffold solutions with different percentages of Mo_132_, after preparation of complete uniform solutions, was measured at 30 °C by the conductivity meter.

### Synthesis of nanofibers containing Mo_132_

The synthesis of [(NH_4_)_42_[Mo_132_O_372_(CH_3_COO)_30_(H_2_O)_72_]∙300H_2_O (Mo_132_) was performed according to the literature^31^. Red–brown crystals of Mo_132_ (3.3 g) were synthesized after a week using hydrazine (0.8 g), ammonium heptamolybdate tetrahydrate (5.6 g) and ammonium acetate (12.5 g) in deionized water (250 mL) and then acetic acid (50%, 83 mL). In the next step, CTAB (0.109 g, 0.3 mmol), TBAB (0.097 g, 0.3 mmol) and MTN (0.428 g) were added into chloroform (20 mL), and Mo_132_ (according to Table S1: 0, 83, 166, or 332 mg for NF1, NF2, NF3, and NF4, respectively) was added into deionized water (10 mL). In the next step, the organic phase was added into the solution of Mo_132_ POM with stirring. After three hours, the mixture was centrifuged at 10,000 rpm for 15 min and the precipitate washed by deionized water (4 times). Finally, the precipitate was dried at 40 °C and the resulted powder was solved into chloroform (1 mL). BPO (20 mg) was added into MMA (1 mL) for polymerization and the resulted solution was added to the first container. The resulted mixture was kept in oven at 70 °C for three hours.

In order to prepare the nanofibers by electrospinning process, the fabricated gel with some amount of solvent (chloroform) was shaken to become sol. For the electrospinning process, the resulted sol was filled in a 1 mL syringe with a 23 G blunted stainless-steel needle using a syringe pump. The solution was injected from a 1 mL syringe with 23 G blunted stainless-steel needle using a syringe pump at flow rate of 1 mL/h. Optimized high voltage (16–18 kV) was applied between the needle and rotating collector, which is laid with aluminum foil at a rotating rate of 80 rpm. The distance between the needle and the collector was 18 cm and kept constant during the electrospinning process.

### Surface hydrophilicity

The hydrophilicity of the MTN-loaded PMMA/Mo_132_ nanofibers was evaluated using water contact angle measurements (n = 3) by the image J software contact angle analyzer. A distilled water droplet size of ~ 5 µL from a syringe was placed carefully on the surface of membranes at room temperature. After a period of 10 s, the contact angle was recorded.

### Water uptake properties

To calculate the amount of water uptake, the pre-weighted membranes (W_0_) were dipped in deionized water (T = 37 °C) for about one hour to completely swell. Then, the samples were removed, and the excess water was wiped off and weighted (W_d_). The amount of water uptake (n = 3) was calculated using following equation^43^:

$$ {\text{Water}}\;{\text{uptake}}\;{\text{( \% ) = }}\frac{{{\text{W}}_{d} - {\text{W}}_{0} }}{{{\text{W}}_{0} }} \times {100}{\text{.}}$$

### Drug/POM encapsulation efficiency and drug release behavior

A known mass of nanofibers was dissolved in DMSO (3 mL) to determine the drug or POM encapsulation efficiency of the nanofibers. The solution was centrifuged and then the liquid supernatant was detected by ultraviolet–visible spectrophotometer (UV–Vis; V-630, JASCO, Japan) at an optimal wavelength of 318 nm. This process was also conducted for nanofibers without MTN or Keplerate POM in the same weight to eliminate any unpredictable absorbance of other contents. The amount of MTN and Mo_132_ were obtained from their calibration curves. The encapsulation efficiency was calculated using the following equation:

$$ {\text{EE}}\;{\text{( \% ) = }}\frac{{{\text{weight}}\;{\text{of}}\;{\text{drug}}\;{\text{or}}\;{\text{POM}}\;{\text{in}}\;{\text{the}}\;{\text{sample}}\;{\text{(g)}} \times {100}}}{{{\text{theoretical}}\;{\text{weight}}\;{\text{of}}\;{\text{loaded}}\;{\text{drug}}\;{\text{or}}\;{\text{POM}}\;{\text{in}}\;{\text{the}}\;{\text{sample}}\;{\text{(g)}}}}.$$

To determine the drug release profiles, fibers were cut into circles with 2 cm in diameter, accurately weighed, and immersed in 5 mL of phosphate buffer saline (PBS) solution (pH 7.4), and then placed in water bath at 37 °C. Then, 1 mL of PBS solution were withdrawn and analyzed by UV–Vis at optimal wavelength of 318 nm at selected predetermined time intervals. The remaining solution was removed and replaced with another 5 mL of fresh PBS. The amount of released drug was determined from the calibration curve of MTN in PBS using UV–Vis spectrophotometer.

### In-vitro biodegradation and bioactivity

To obtain the biodegradation profile of the membranes, they were cut into circular samples of 2 cm in diameter, accurately weighed (W_0_), and immersed in 5 mL of PBS at 37 °C. Then, samples were carefully removed at selected predetermined times, washed with deionized water three times, completely dried at 40 °C and weighed again (W_d_). The weight loss of each sample was calculated according to the following equation:

$$ {\text{Weight}}\;{\text{loss}}\;{\text{( \% )}} = { }\frac{{{\text{W}}_{0} - {\text{W}}_{d} }}{{{\text{W}}_{0} }} \times {100}{\text{.}}$$

The in-vitro bioactivity of membranes was characterized by soaking them in simulated body fluid (SBF) solution with the pH of 7.4 at a constant temperature of 37 °C for 28 days. The SBF was prepared using the method developed by Kokubo and Takadama^44^. Then, samples were extracted and dried at room temperature for 24 h. The morphology of dried samples was characterized by SEM and the chemical composition of apatite layer on the surface of membranes was confirmed by FT-IR analysis.

### Biocompatibility assessment: cytotoxicity assays

To assess the cell viability, MTT assay was used according to the manufacturer’s instructions. After 1, 3 and 7 days of culture, the samples were presoaked in Dulbecco's Modified Eagle Medium (DMEM) and MTT (1:1) solutions at 37 °C under CO_2_ atmosphere (5%) for 3 h. Then, DMSO (5 mL) was added to the solution to dissolve the purple formazan crystals. Finally, 100 µL of the solution was transferred to 96-well culture plates and optical density was measured at 570 nm using an ELISA Reader (Stat Fax-2100; GMI, Inc., Miami, FL, USA).

### Cell culture

To enable cell seeding, each sample was cut into a circle with 1.5 cm in diameter, washed with phosphate-buffered saline (PBS), exposed under ultraviolet light for 20 min and put into the 24-well plate. Human osteoblast-like cells, MG-63, from National Cell Bank of Iran at the Pasteur Institute were re-suspended in Dulbecco’s Modification of Eagles Medium (DMEM; GIBCO, Scotland) supplemented with 10% (v/v) Fetal Bovine Serum (FBS; Gibco, Renfrewshire, Scotland) and 1% (v/v) penicillin (Sigma, Saint Louis, USA)/streptomycin (Sigma, Saint Louis, USA) at 37 °C in a humidified incubator with 5% CO_2_. The culture medium was refreshed every 3 days.

### Cell morphology observation

The morphology of MG-63 cells was observed using SEM after 7-days culture with the samples (2 × 104 cells/cm^2^). To achieve this, samples were washed with PBS and the cells were fixed by soaking in a solution of glutaraldehyde (2.5%) in PBS (0.1 M) for 3 h at 4 °C, and then post-fixed with osmium tetroxide (0.1%) in PBS (0.1 M) for 30 min^45^. Afterwards, the samples were washed with PBS, dehydrated by graded ethanol (30%, 70%, 90%, 95%, and 100% ethanol; each step 10 min), and finally dried at room temperature.

### Antibacterial activity of nanofiber scaffolds

In order to measure the qualitative antibacterial performance of nanofiber scaffolds, the agar diffusion method was used against the Gram-negative Escherichia coli. Along this, the antibacterial activity of NF4 samples was carried out by exposing disc shaped specimens (1 cm diameter) to Escherichia coli and the zone of inhibited bacterial growth around the specimens was monitored. In this regard, the specimens were applied on the bacterial-seeded Mueller Hinton Agar plate (1.0 × 108 CFU/mL) and the inhibition zone of the samples was visually investigated after 24 h of incubation at 37 °C.

## Results and discussion

### Chemical, thermal and mechanical properties of nanofiber scaffolds

**Figure 2:**
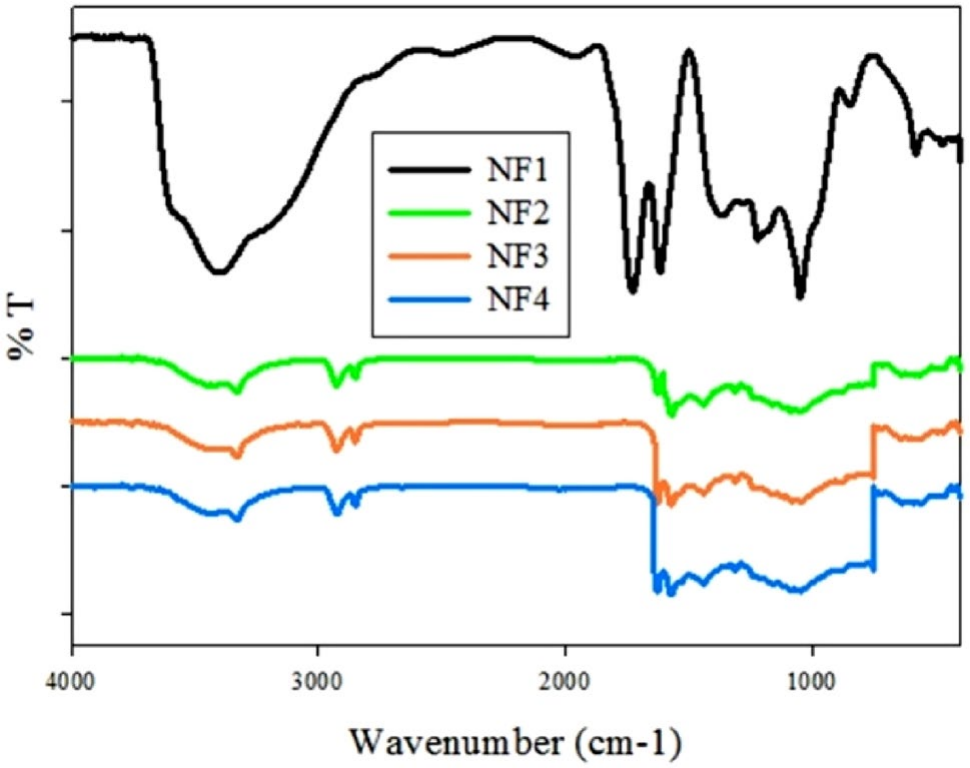
The FT-IR spectra of NF1, NF2, NF3, and NF4.

The chemical structure of nanofiber scaffolds was studied by FT-IR to evaluate the chemical interaction between MTN and POM. As shown in Fig. 2, the FT-IR spectrum of PMMA nanofiber scaffolds presents the characteristic bands of PMMA. Three main absorption bonds of PMMA are at 2850–2815 (O–CH_3_), 1650–1790 (–C=O) and 1430–1470 cm^−1^ (−CH_3_)^46^. On the other hand, it was confirmed that the electrospinning process did not alter the molecular structure of MTN, particularly, their antibacterial NO_2_ group. Absorption bands at 1535 and 1366 cm^−1^ are attributed to the MTN NO_2_ group^47^. In Fig. 1, additional bands for NF4 at approximately 1546 (m, COO), 1407 (m), 936 (vs), 792 (m), 723, and 567 (s) cm^−1^ belong to Mo_132_ structure^31^. These findings indicate that the molecular structure of nanofibers does not change by the electrospinning process. Moreover, the FT-IR spectrum of NF1 does not show 1546, 1407, 936, 792, 723, and 567 cm^−1^ bonds due to the lack of Mo_132_. The FT-IR spectrum of NF2 and NF3 also clearly displays the existence of PMMA, Mo_132_ and MTN in the nanofiber scaffolds.

The TGA diagrams for NF1, NF2, NF3, and NF4 nanofibers are similar (Fig. S1). In these TGA diagrams the weight losses at 150 to 210 °C correspond to the decomposition of MTN and other organic groups. NF4 and NF1 with the highest and lowest amount of Mo_132_ respectively show the highest and lowest degradation at 180 to 230 °C temperature range. In the Mo_132_, the first weight loss is relating to the acetate ligands of structure. Moreover, the PMMA and POM have weight losses around 380 °C. Accordingly, the TGA analysis confirms hybridization of PMMA, MTN, and POM.

Figure S2 shows the XRD pattern of POM/MTN incorporated PMMA. Two diffraction peaks at about 18° and 50° are characteristic peaks of semi-crystalline PMMA. For MTN incorporated PMMA, the relative intensity of characteristic peaks decreased due to the decrease in PMMA crystallinity. Also, these two peaks have been shifted slightly to the lower angles, probably because of the interaction between PMMA, POM and MTN, or the trapping of Mo_132_ and MTN molecules among the PMMA chains. Characteristic diffraction peaks of MTN at 12.2° and 13.8° confirm the aggregation of MTN.

**Figure 3:**
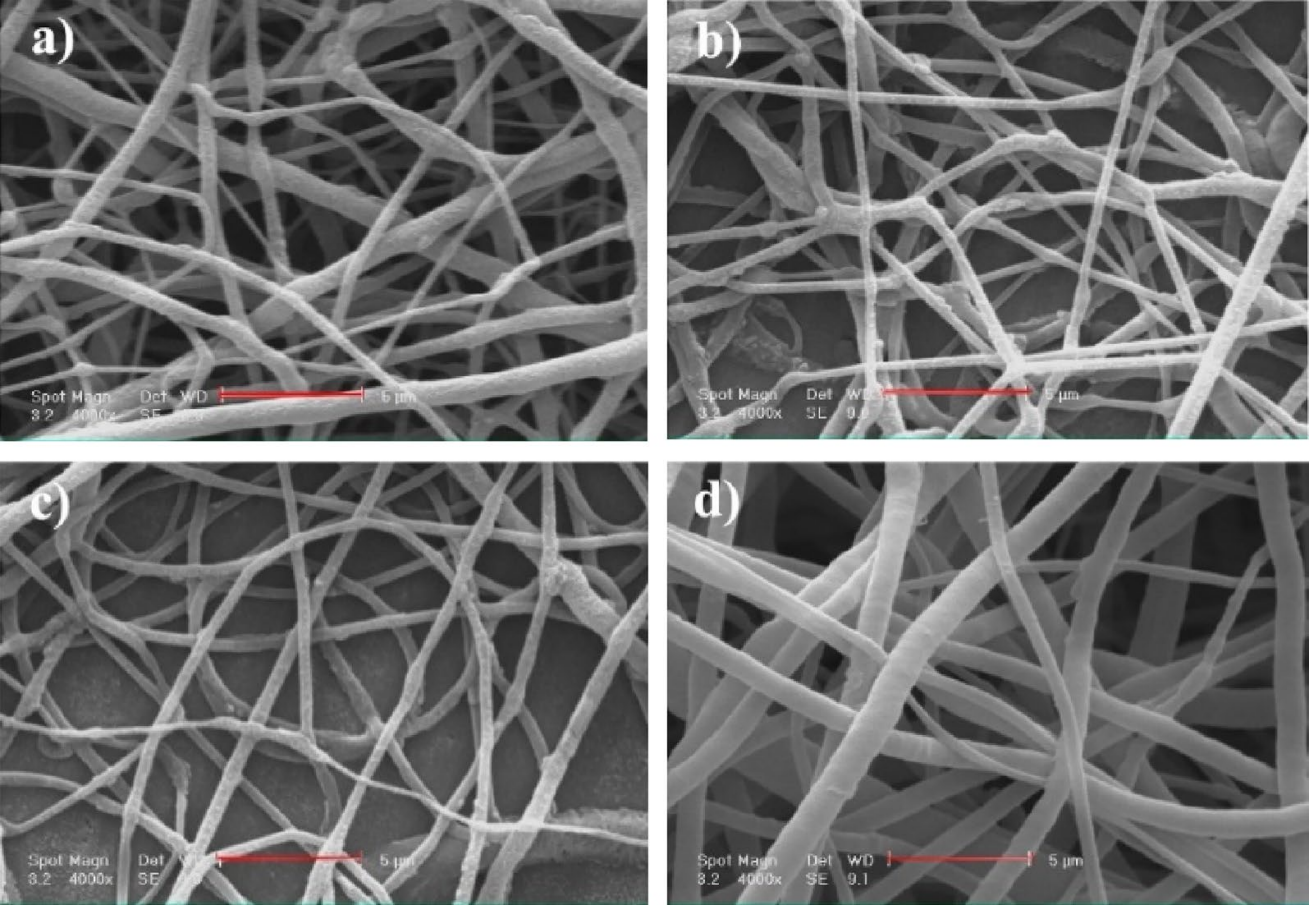
SEM images of NF1 (**a**), NF2 (**b**), NF3 (**c**), and NF4 (**d**) (scale bar: 5 µm).

The surface morphology of NF1, NF2, NF3, and NF4 as well as their fiber diameter distribution are shown in Fig. 3. Fiber diameters, electric conductivity of different electrospinning solutions, and porosity values of different nanofiber scaffolds are also listed in Table S1. As shown in Fig. 3, NF1 displays a randomly interconnected structure with smooth morphology and uniform distribution of electrospun bead-free nanofiber scaffolds. Electrical conductivity of the spinning solution was increased by addition of POM, while the fiber's diameter and porosity of the nanofiber scaffolds were decreased (Table S1). With increase in Mo_132_ dosage, the average fiber diameter reduced from 445 ± 99 nm to 368 ± 99 nm. Generally, a large number of electric charges are carried by the electrospinning jet. Thus, the electrical field supports more elongation forces on the jet. Consequently, the jet path became longer, and the fiber diameters have decreased^48^. Conductivity plays a significant role in this study when CTAB, TBAB and POM are incorporated into the nanofiber scaffolds. Thus, fiber diameter is decreased with increase in POM amounts. However, for NF4, the increase is possibly due to aggregation of POM. The porosity in the range of 60–90% is sufficient for GTR membrane to ensure nutrient exchange^49^. According to Table S1, the surface porosity of nanofiber scaffolds is reduced with increase in POM contents, which is attributed to the reducing in fiber diameters of the nanofibers. In fact, fibers with smaller diameter sizes overlaid with each other, and thus, lower porosity could be achieved by filling the pores^43^. Hosseini et al. declared that reduction in fiber diameters of scaffolds can cause the lower surface porosity of scaffolds, which is aligned with achieved results^50^.

**Figure 4:**
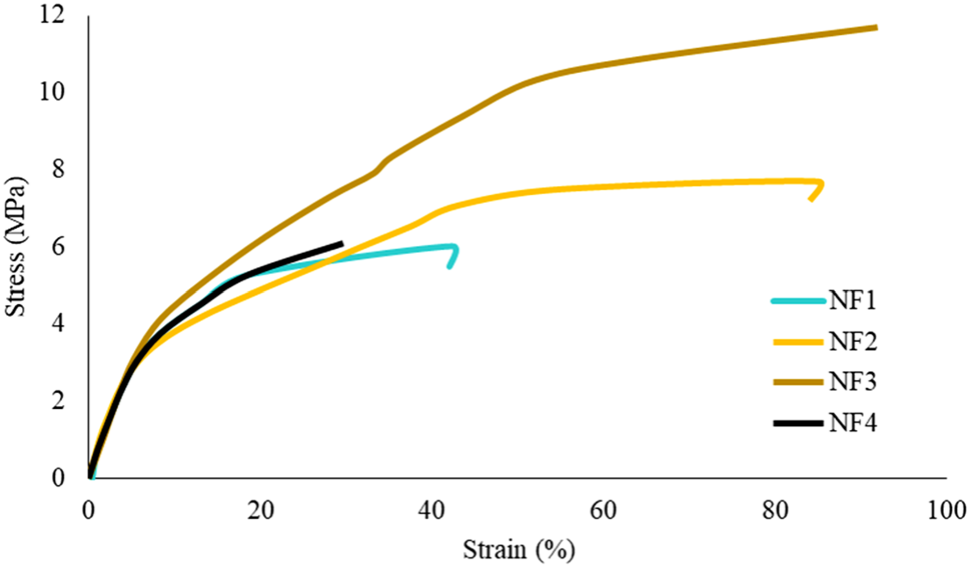
The stress–strain curves of NF1, NF2, NF3, and NF4.

Mechanical properties of nanofiber scaffolds are among important physical properties of GTR/GBR. The GTR/GBR applications must sustain adequate mechanical properties during the surgical procedures. Figure 4 displays the typical stress versus strain response of the nanofibers. As it could be seen, tensile strength of nanofibers varies from 6 ± 1 to 11.7 ± 3.2 MPa. The Mo_132_ contents affect the tensile strength and elongation at breaking point of the nanofibers. With increase in Mo_132_ content, the tensile strength and elongation at break point also increase. For instance, the tensile strength for NF1 is improved from 6 ± 1 MPa to 7.7 ± 3.2 MPa by addition of Mo_132_ to the PMMA polymer. Generally, incorporation of PMMA polymers and inorganic particles, such as POMs, into organic polymers can improve the mechanical properties, e.g., stiffness and strength of polymers^51^. Also, similar studies reported the enhancement of mechanical properties in nanocomposites containing POMs compared to pure polymer^52^. Interaction and adhesion between POM and PMMA could enhance their tensile strength. Additionally, Mo_132_ and MTN can function as fillers in the polymer matrix which increases the hardness and stiffness of nanofiber scaffolds. Table S2 summarized the ultimate tensile strength, elastic modulus, and elongation at the break point of the fabricated nanofiber. These results suggest that the mechanical properties of the pure PMMA nanofibers could be improved by incorporating different contents of Mo_132_. However, a higher Mo_132_ content (NF4) significantly affects the desired mechanical properties in a negative way. This effect is caused by the agglomeration of nanoparticles in nanofiber scaffolds.

### Hydrophilicity of nanofiber scaffolds

The hydrophilicity of the membrane has a significant effect on the adhesion and proliferation of cells^53^. The PMMA suffers from low hydrophilicity despite having good biocompatibility. To assess the effect of MTN and Mo_132_ on the hydrophilicity of nanofiber scaffolds, the water contact angles were measured and the shape of water droplet on the surface of nanofiber scaffolds was observed. Moreover, the water uptake of scaffolds was calculated according to the related Equation. Table S3 presents the water contact angles and water uptake of various nanofiber scaffolds. Based on PMMA and POM contents, the water uptakes and contact angles for NF1 to NF4 nanofiber scaffolds show improvement from 14.18 ± 0.62% to 35.62 ± 0.24% and from 126 ± 5.2° to 83.9 ± 3.2°, respectively. The incorporation of hydrophilic MTN can improved the hydrophilicity of nanofibers. This corresponds to the polar hydroxyl and imidazole groups on the MTN molecule^54^. The presence of charged POM (Mo_132_) also improves the hydrophilicity of nanofiber scaffolds^55^. Results in Table S3 represent the enhanced nano-composition hydrophilicity by incorporation of POM and MTN.

### Drug encapsulation efficiency and drug release behavior

The drug encapsulation efficiencies for different amounts of Mo_132_ and MTN are listed in Table 1. Based on the results in Table 1, all samples show sufficient drug encapsulation efficiencies because of the good drug dispersion in nanofibers. Also, this is presented by XRD pattern and FT-IR spectra.

**Table 1:** Encapsulation efficiency (EE) for different amounts of Mo_132_ and MTN.

Sample	Mo_132_ (mg)	MTN (mg)	Mo_132_ EE (%)	MTN EE (%)
NF1	0	428	–	85
NF2	83^a^	428	74 ± 4	68 ± 3
NF3	166	428	71 ± 3	76 ± 4
NF4	332	428	75 ± 3	81 ± 3

^a^3 µmol.

**Figure 5:**
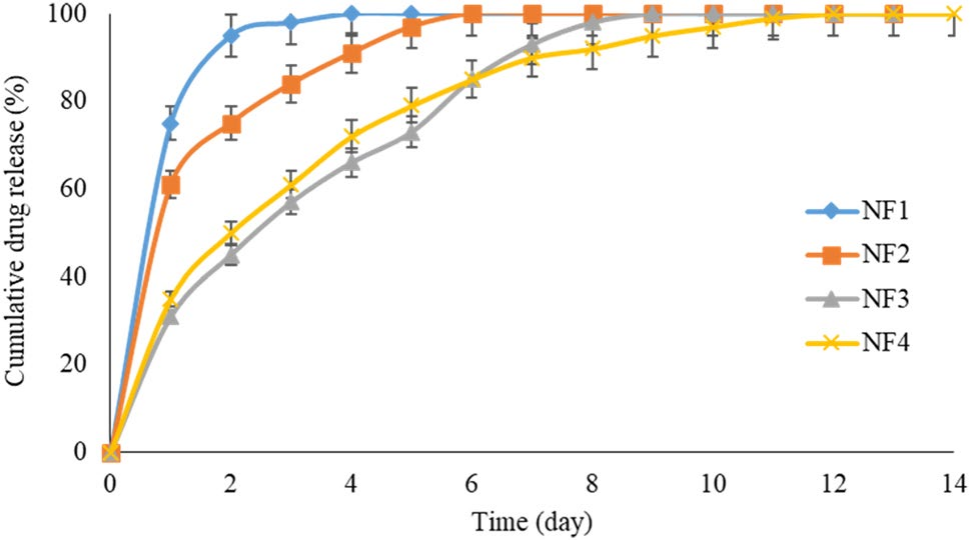
Drug release behavior of NF1, NF2, NF3, and NF4 in PBS buffer for 14-days.

The drug release behavior of samples in PBS buffer solution was estimated over a 14-days period. Figure 5 shows the cumulative drug release behavior of these nanofiber scaffolds. The release of around 70 percent of drugs within 7 days indicates the adequate drug release after implantation of the scaffold on the defect site. The first week after implantation is a susceptible period of infection. Thus, high initial drug release is suggested to eliminate bacteria in a few days after the implant^56^.

During the second week of drug release, the MTN was released in a linear way. The drug solubility and interactions in polymer/drug/POM could affect the encapsulation and drug release in the nanofibers^57^. However, although PMMA is hydrophobic and MTN and Mo_132_ are hydrophilic, they can form strong hydrogen bonds. Moreover, Mo_132_ can form ionic bonds with MTN molecules^58^. The higher contents of Mo_132_ in nanofibers show a lower MTN release. This is probably attributed to the existence of hydrogen and oxygen groups in Mo_132_ which could form hydrogen bonds with CN, OH, and NO_2_ groups of MTN molecules. Based on the above explanations, the drug release behavior has lasted for more than 10 days.

### In-vitro biodegradation and bioactivity of nanofiber scaffolds

**Figure 6:**
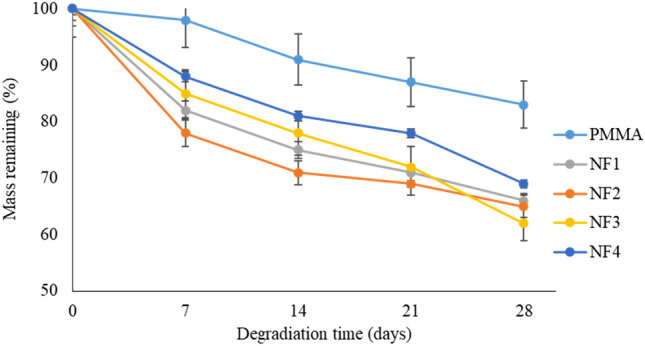
In-vitro degradation of NF1, NF2, NF3, and NF4 for 28 days.

To evaluate the in-vitro biodegradability of NF1 to NF4 nanofiber scaffolds, their weights were plotted against interval times for 28 days of immersing in PBS at 37 °C. The results (Fig. 6) do not show significant mass lose for pure PMMA nanofiber scaffolds in the 28-day period. Nevertheless, a fast mass loss was observed for NF2, NF3, and NF4 during 15 days of incubation, mainly due to the drug release mass. Also, further mass losses of NF2, NF3 and NF4 were observed with increase in Mo_132_ contents. This could be attributed to POM and MTN hydrophilic groups that could accelerate the penetration of water into the nanofiber scaffolds and subsequent hydrolysis of PMMA.

**Figure 7:**
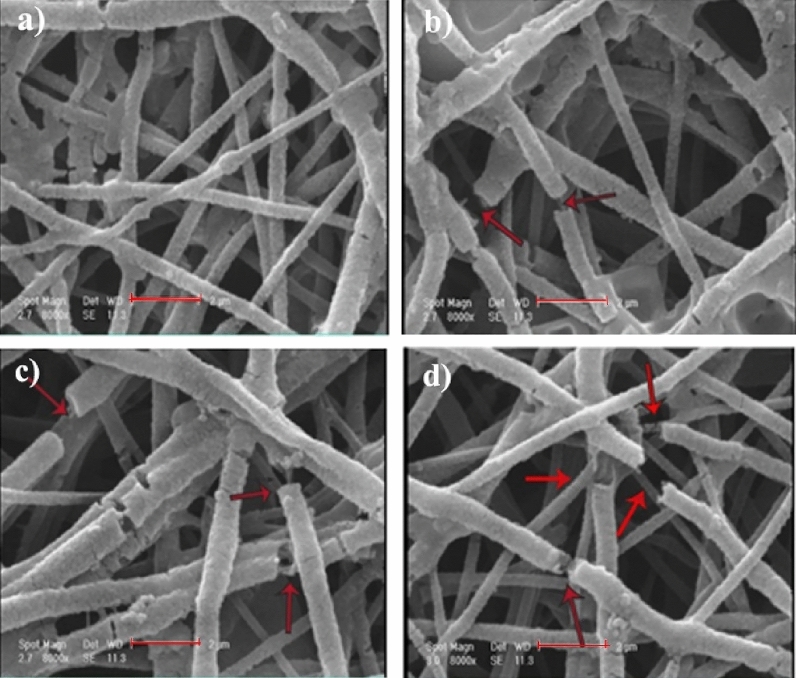
SEM images of NF1 (**a**), NF2 (**b**), NF3 (**c**), and NF4 (**d**) in PBS solution after 28 days (scale bar: 5 µm).

The red arrows in Fig. 7 indicate the cracks and voids on the surface of nanofibers after 28 days’ immersion in PBS. While the morphology of PMMA scaffold shows a slight change, the surface morphology of NF2, NF3, and NF4 indicates an obvious alteration with increase in Mo_132_ contents. Moreover, it should be noted that low PMMA degradation rate may limit its biomedical applications. This problem has been solved innovatively in the present study by incorporation of hydrophilic MTN and Mo_132_ into the PMMA nanofibers.

**Figure 8:**
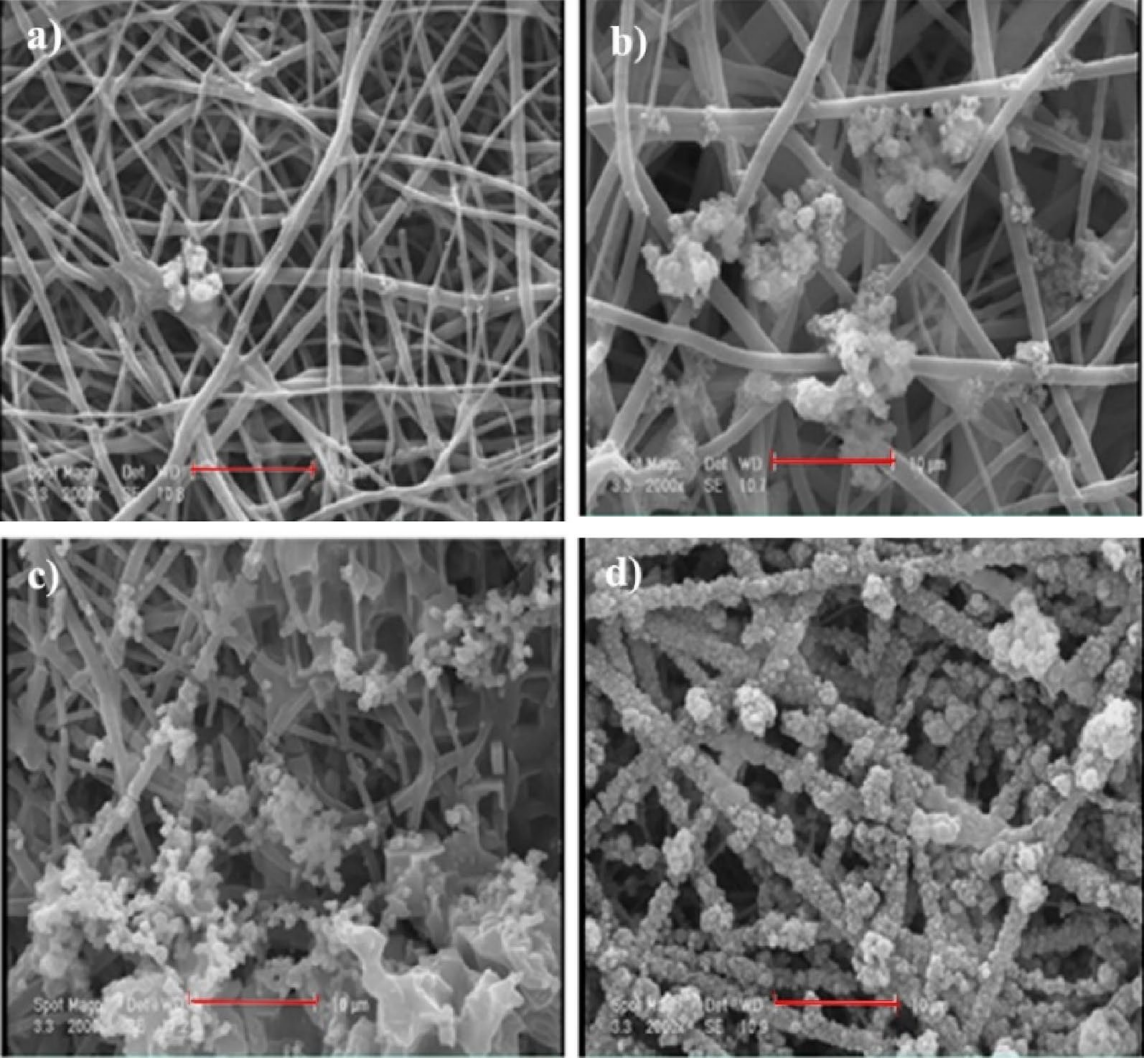
SEM images of NF1 (**a**), NF2 (**b**), NF3 (**c**), and NF4 (**d**) in SBF solution after 28 days (scale bar: 10 µm).

The in-vitro bioactivity behavior of NF1, NF2, NF3, and NF4 scaffolds was evaluated by soaking these nanofiber scaffolds in SBF solution. Figure 8 shows the SEM images of different scaffolds immersed in SBF solution after 28 days. The NF2, NF3, and NF4 scaffolds show excellent ability to form a hydroxyapatite layer compared to NF1. As shown in Fig. 8, the hydroxyapatite layer formation is potentially enhanced with increase in Mo_132_. This happens because the terminal oxygen groups of Mo_132_ could aggregate with P_2_O_5_ and CaO from simulated body fluid solution. FT-IR was used to confirm the presence of hydroxyapatite on the nanofiber scaffolds (Fig. S3). The PO_4_^−3^ bands of hydroxyapatite group appear at 560, 604 and 1048 cm^−1^^59^.

### Biocompatibility assessment of nanofiber scaffolds

**Figure 9:**
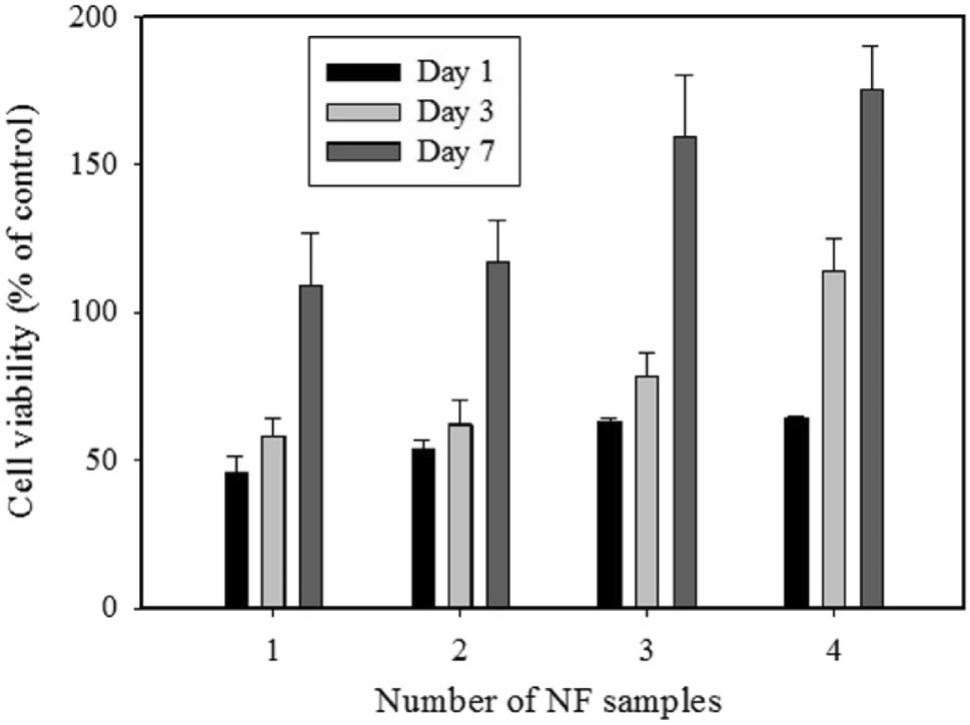
Cell viability (MTT assay) on NF1, NF2, NF3, and NF4 after 1, 3, and 7 days.

The cell proliferation was evaluated after 1-, 3-, and 7-days of culturing with NF1, NF2, NF3, and NF4 using MTT assay (Fig. 9). No significant difference was observed in the cell viability after 1-day incubation with nanofiber scaffolds containing different Mo_132_ contents. After 3 days, nanofiber scaffolds with the highest Mo_132_ content presented a higher absorbance value in comparison to the control group (p < 0.05). This could be attributed to the gradual release of POM from the scaffolds, which could stimulate the proliferation of cells^60^.

**Figure 10:**
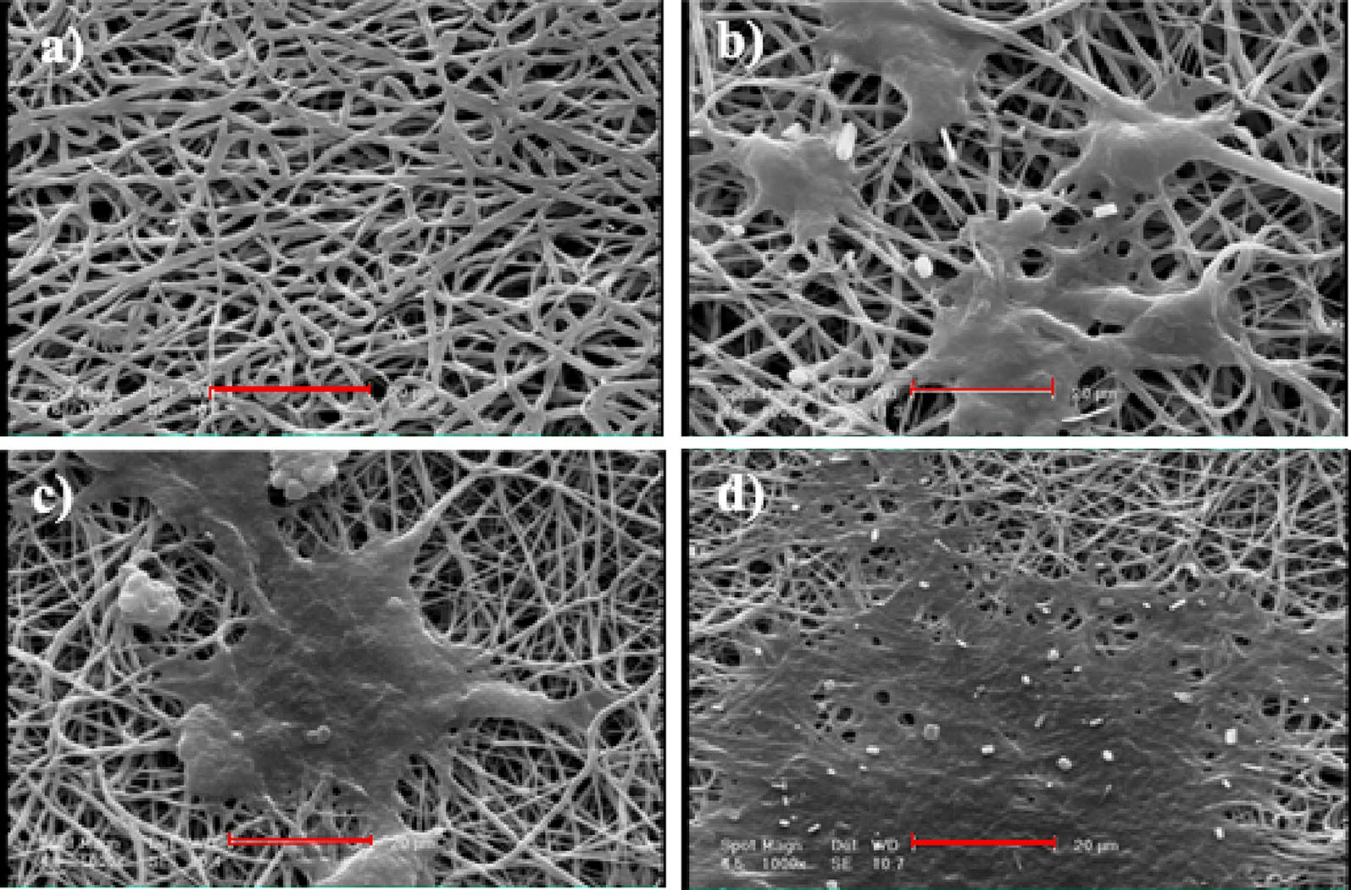
NF1 (**a**), NF2 (**b**), NF3 (**c**), and NF4 (**d**) morphology of the 7-days cultured MG-63 cells (scale bar: 20 µm).

Interestingly, after 7 days of culture, the proliferation of cells incubated with different samples was significantly improved with increase in Mo_132_ concentration. The morphology of cultured MG-63 cells was observed by SEM after 7-day culture on the scaffolds (Fig. 10). As presented in Fig. 10, the number of MG-63 cells adhered to nanofiber scaffolds showed considerable increase at higher amounts of Mo_132_. This could be attributed to the hydrophilicity of scaffolds. Moreover, these images clearly demonstrate the proper proliferation of MG-63 cells and no toxic effect of Mo_132_ on them.

### Antibacterial assay of nanofiber scaffolds

As stated above, the qualitative antibacterial performance of nanofiber scaffolds was measured using the agar diffusion method. As it could be seen in Fig. S4, a significant inhibition zone around the NF4 specimens is observe against Escherichia coli bacteria. Moreover, a little inhibition zone is detecting around the MTN free NF4 sample. Also, lesser antibacterial effect is attaining with solely MTN. Therefore, the NF4 antibacterial property is due to the synergistic effect of MTN, Mo_132_ and PMMA.

## Conclusion

In present study, the Mo_132_/MTN/PMMA nanofiber scaffolds for GTR/GBR with controlled drug delivery were fabricated by electrospinning technique. Various contents of Mo_132_ were embedded into PMMA microfibers according to the optimum condition for electrospinning. The Mo_132_ was homogeneously hybridized with the metronidazole-load PMMA polymer, while its intrinsic characteristics were preserved. The incorporation of Mo_132_ into metronidazole-load PMMA resulted in an exceptional reduction in fibers diameter (445 nm to 365 nm) and improved water uptake (14.18% to 35.6%), contact angle (126° to 83.9°), drug encapsulation efficiency, degradation rate, bioactivity, and mechanical properties. However, for NF_4_ sample, decrease in mechanical properties may be due to the agglomeration of nanoparticles at high Mo_132_ content. This system provides the controlled release of MTN for 14 days. Such a prolonged controlled release of the antibacterial drugs can prevent the colonization of anaerobic microorganisms. Cell assessments expose the potential of Mo_132_ contents to support osteoconductivity of nanofiber scaffolds and attachment of MG-63 cells. Moreover, after 7 days of culture, viability assay confirmed that the nanofiber scaffolds containing Mo_132_ obviously improve the cell proliferation with increase in Mo_132_ content compared to pure PMMA nanofiber scaffolds. These findings suggest that Mo_132_ embedded drug-load PMMA micro/nanofibers have excellent potential to function as a membrane for guided bone regeneration.

## Supplementary Information

Supplementary Information.
