# Supplementary material for: Controlled drug delivery and cell adhesion for bone tissue regeneration by Keplerate polyoxometalate (Mo132)/metronidazole/PMMA scaffolds
Source: Sci Rep. 2022 Aug 24;12:14443. doi: 10.1038/s41598-022-18622-w (PMC9402948; doi:10.1038/s41598-022-18622-w)
Supplement: Supplementary file 1 — Supplementary Information. [file 41598_2022_18622_MOESM1_ESM.docx]

**Controlled drug delivery and cell adhesion for bone tissue regeneration by Keplerate polyoxometalate (Mo_132_)/metronidazole/PMMA scaffolds**

Hamid Taghiyar^1^, Bahram Yadollah*^,^1, Abolghasem Abbasi Kajani^2^

^1^*Department of Chemistry, University of Isfahan, Isfahan 81746-73441, Iran*

^2^*Department of Biotechnology,* Faculty of Biological Science and Technolog*y, University of Isfahan, Isfahan 81746-73441, Iran*

**Corresponding Author. Tel: +98-31-37934934; fax: +98-31-36689732; e-mail:* [*yadollahi@chem.ui.ac.ir*](mailto:yadollahi@chem.ui.ac.ir)*,* [*yadollahi.b@gmail.com*](mailto:yadollahi.b@gmail.com)

**Supporting Information**

**Materials and methods**

3-(4,5-dimethylthiazol-2-yl)-2,5-diphenyltetrazolium bromide (MTT; Sigma, Saint Louis, USA), [hydrazine sulfate](https://www.chemicalbook.com/ChemicalProductProperty_EN_CB2679815.htm), [ammonium heptamolybdate tetrahydrate](http://www.cfmot.de/en/ammonium-hepta-molybdat-tetrahydrat.html), [ammonium acetate](https://en.wikipedia.org/wiki/Ammonium_acetate), [acetic acid](https://pubchem.ncbi.nlm.nih.gov/compound/acetic_acid), [tetrabutylammonium bromide](https://www.sigmaaldrich.com/catalog/product/sial/426288?lang=en&region=US) (TBAB), [cetyltrimethylammonium bromide](http://www.chemspider.com/Chemical-Structure.5754.html) (CTAB), methyl methacrylate (MMA), benzoyl peroxide (BPO), and all of the used solvents were purchased from Sigma–Aldrich or some of the other chemical companies. All of the other chemicals were analytical grade and applied without further purification. Metronidazole (MTN) was purchased from Amin pharmacy (Iran).

**Characterization methods**

To confirm the chemical composition of nanofibers, Fourier transform infrared spectroscopy (FT-IR; JASCO FT/IR-680 PLUS) was performed over a range of 400–4000 cm^-1^ and resolution of 2 cm^-1^. Powder X-ray diffraction (XRD) was conducted using X'Pert Pro X-ray diffractometer (Phillips, Netherlands) with CuK" radiation (k = 0.15406 nm) at a generator voltage of 40 kV and a current of 40 mA. The thermal stability of nanofibers was investigated by thermogravimetric analysis (TGA; Rheometric scientific 1998, USA). The nanofibers were heated from 30 to 800 °C at a heating rate of 10 °C/min and the weight losses of samples during the test were used for calculations. The surface morphology of nanofibers was observed by scanning electron microscopy (SEM) analysis using a Philips XL30 SEM. The nanofibers were coated with a thin layer of gold before being observed under the microscope and fiber diameter size was measured using the Image J software on SEM micrographs at 20 random locations. The surface areas were calculated using the BET equation and pore size distribution curves were calculated via the BJH method. The pore volume was estimated to be up to P/P_0_ = 0.98.

The mechanical properties of various nanofibers were evaluated by tensile strength tests (INSTRON, Zwick, The United Kingdom) with 10 N load capacity at a rate of 10 mm/min. Samples with dimensions of 70 mm × 10 mm were prepared in rectangular pieces. At least 3 samples were prepared for each nanofiber composition. Tensile strength, tensile modulus, and strain at break were determined from the stress–strain curve. Tensile modulus was determined from the slope of initial linear portion of stress-strain curve while strain at break was obtained when samples failed. Samples were evaluated to obtain the mean and standard deviation (SD) for each nanofibrous. Electrical conductivity was measured by Schott conductivity meter (CG885). The electrical conductivity of the scaffold solutions with different percentages of Mo_132_, after preparation of complete uniform solutions, was measured at 30 °C by the conductivity meter.

**Synthesis of Mo_132_ containing nanofibers**

The synthesis of [(NH_4_)_42_[Mo_132_O_372_(CH_3_COO)_30_(H_2_O)_72_]∙300H_2_O (Mo_132_) was performed according to the literature[^1^](#_ENREF_30). Red-brown crystals of Mo_132_ (3.3 g) were synthesized after a week using hydrazine (0.8 g), ammonium heptamolybdate tetrahydrate (5.6 g) and [ammonium acetate](https://en.wikipedia.org/wiki/Ammonium_acetate) (12.5 g) in deionized water (250 mL) and then acetic acid (50%, 83 mL). In the next step, CTAB (0.109 g, 0.3 mmol), TBAB (0.097 g, 0.3 mmol) and MTN (0.428 g) were added into chloroform (20 mL), Mo_132_ (according to Table 1: 0, 83, 166, or 332 mg for NF1, NF2, NF3, and NF4, respectively) was added into deionized water (10 mL). In the next step, the organic phase was added into the solution of Mo_132_ POM with stirring. After three hours, the mixture was centrifuged at 10000 rpm for 15 min and the precipitate washed by deionized water (4 times). Finally, the precipitate was dried at 40 °C and the resulted powder was solved into chloroform (1 mL). BPO (20 mg) was added into MMA (1 mL) for polymerization and the resulted solution was added to the first container. The resulted mixture was kept in oven at 70 °C for three hours.

In order to prepare the nanofibers by electrospinning process, the fabricated gel with some amount of solvent (chloroform) was shaken to become sol. For the electrospinning process, the resulted sol was filled in a 1 mL syringe with a 23 G blunted stainless-steel needle using a syringe pump. The solution was injected from a 1 mL syringe with 23 G blunted stainless-steel needle using a syringe pump at flow rate of 1 mL/h. Optimized high voltage (16-18 kV) was applied between the needle and rotating collector, which is laid with aluminum foil at a rotating rate of 80 rpm. The distance between the needle and the collector was 18 cm and kept constant during the electrospinning process.

**Surface hydrophilicity**

The hydrophilicity of the MTN-loaded PMMA/Mo_132_ nanofibers was evaluated using water contact angle measurements (n = 3) by the image J software contact angle analyzer. A distilled water droplet size of ∼ 5 𝜇L from a syringe was placed carefully on the surface of membranes at room temperature. After a period of 10 seconds, the contact angle was recorded.

**Water uptake properties**

To calculate the amount of water uptake, the pre-weighted membranes (W_0_) were dipped in deionized water (T = 37 °C) for about one hour to completely swell. Then, the samples were removed, and the excess water was wiped off and weighted (W_d_). The amount of water uptake (n = 3) was calculated using equation 1[^2^](#_ENREF_42):

| $\text{Water uptake (\%) = }\frac{\text{W}_{d}\text{-}\text{W}_{0}}{\text{W}_{0}}\text{×100}$ | (Eq. 1) |
| --- | --- |

**Drug/POM encapsulation efficiency and drug release behavior**

A known mass of nanofibers was dissolved in DMSO (3 mL) to determine the drug or POM encapsulation efficiency of the nanofibers. The solution was centrifuged and then the liquid supernatant was detected by ultraviolet-visible spectrophotometer (UV-vis; V-630, JASCO, Japan) at an optimal wavelength of 318 nm. This process was also conducted out for nanofibers without MTN or Keplerate POM in the same weight to eliminate any unpredictable absorbance of other contents. The amount of MTN and Mo_132_ were obtained from their calibration curves. The encapsulation efficiency was calculated using the following equation:

| $\text{EE (\%) = }\frac{\text{weight of drug or POM in the sample (g)}}{\text{theoretical weight of loaded drug or POM in the sample (g)}}\text{ × 100}$ | (Eq. 2) |
| --- | --- |

To determine the drug release profiles, fibers were cut into circles with 2 cm in diameter, accurately weighed, and immersed in 5 mL of phosphate buffer saline (PBS) solution (pH 7.4), and then placed in water bath at 37 °C. Then, 1 mL of PBS solution were withdrawn and analyzed by UV-vis at optimal wavelength of 318 nm at selected predetermined time intervals. The remaining solution was removed and replaced with another 5 mL of fresh PBS. The amount of released drug was determined from the calibration curve of MTN in PBS using UV-vis spectrophotometer.

***in-vitro* biodegradation and bioactivity**

To obtain the biodegradation profile of the membranes, they were cut into circular samples of 2 cm in diameter, accurately weighed (W_0_), and immersed in 5 mL of PBS at 37 °C. Then, samples were carefully removed at selected predetermined times, washed with deionized water three times, completely dried at 40 °C and weighed again (W_d_). The weight loss of each sample was calculated according to equation 3:

| $\text{Weight loss (\%) = }\frac{\text{W}_{0}\text{-}\text{W}_{d}}{\text{W}_{0}}\text{ × 100}$ | (Eq. 3) |
| --- | --- |

The *in-vitro* bioactivity of membranes was characterized by soaking them in simulated body fluid (SBF) solution with the pH of 7.4 at a constant temperature of 37 °C for 28 days. The SBF was prepared using the method developed by Kokubo and Takadama[^3^](#_ENREF_43). Then, samples were extracted and dried at room temperature for 24 h. The morphology of dried samples was characterized by SEM and the chemical composition of apatite layer on the surface of membranes was confirmed by FT-IR analysis.

**Biocompatibility assessment - cytotoxicity assays**

To assess the cell viability, MTT assay was used according to the manufacturer’s instructions. After 1, 3 and 7 days of culture, the samples were presoaked in Dulbecco's Modified Eagle Medium (DMEM) and MTT (1:1) solutions at 37 °C under CO_2_ atmosphere (5%) for 3 h. Then, DMSO (5 mL) was added to the solution to dissolve the purple formazan crystals. Finally, 100 µL of the solution was transferred to 96-well culture plates and optical density was measured at 570 nm using an ELISA Reader (Stat Fax-2100; GMI, Inc., Miami, FL, USA).

**Cell culture**

To enable cell seeding, each sample was cut into a circle with 1.5 cm in diameter, washed with phosphate-buffered saline (PBS), exposed under ultraviolet light for 20 min and put into the 24-well plate. Human osteoblast-like cells, MG-63, from National Cell Bank of Iran at the Pasteur Institute were re-suspended in Dulbecco’s Modification of Eagles Medium (DMEM; GIBCO, Scotland) supplemented with 10% (v/v) Fetal Bovine Serum (FBS; Gibco, Renfrewshire, Scotland) and 1% (v/v) penicillin (Sigma, Saint Louis, USA)/streptomycin (Sigma, Saint Louis, USA) at 37 °C in a humidified incubator with 5% CO_2_. The culture medium was refreshed every 3 days.

**Cell morphology observation**

The morphology of MG-63 cells was observed using SEM after 7-days culture with the samples (2 × 104 cells/cm^2^). To achieve this, samples were washed with PBS and the cells were fixed by soaking in a solution of glutaraldehyde (2.5%) in PBS (0.1 M) for 3 h at 4 °C, and then post-fixed with osmium tetroxide (0.1%) in PBS (0.1 M) for 30 min^4^. Afterwards, the samples were washed with PBS, dehydrated by graded ethanol (30%, 70%, 90%, 95%, and 100% ethanol; each step 10 min), and finally dried at room temperature.

**Antibacterial activity of nanofiber scaffolds**

In order to measure the qualitative antibacterial performance of nanofiber scaffolds, the agar diffusion method was used against the Gram-negative Escherichia coli. Along this, the antibacterial activity of NF4 samples was carried out by exposing disc shaped specimens (1 cm diameter) to Escherichia coli and the zone of inhibited bacterial growth around the specimens was monitored. In this regard, the specimens were applied on the bacterial-seeded Mueller Hinton Agar plate (1.0 × 108 CFU/mL) and the inhibition zone of the samples was visually investigated after 24 h of incubation at 37 °C.


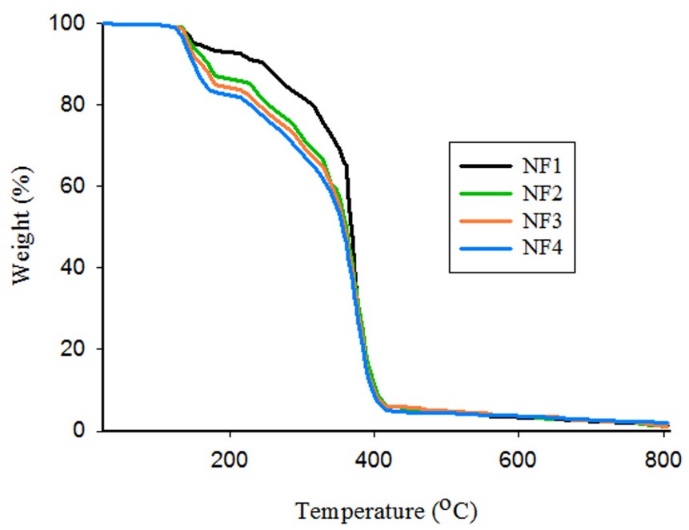


**Figure S1.** The TGA diagram of NF1, NF2, NF3, and NF4

**
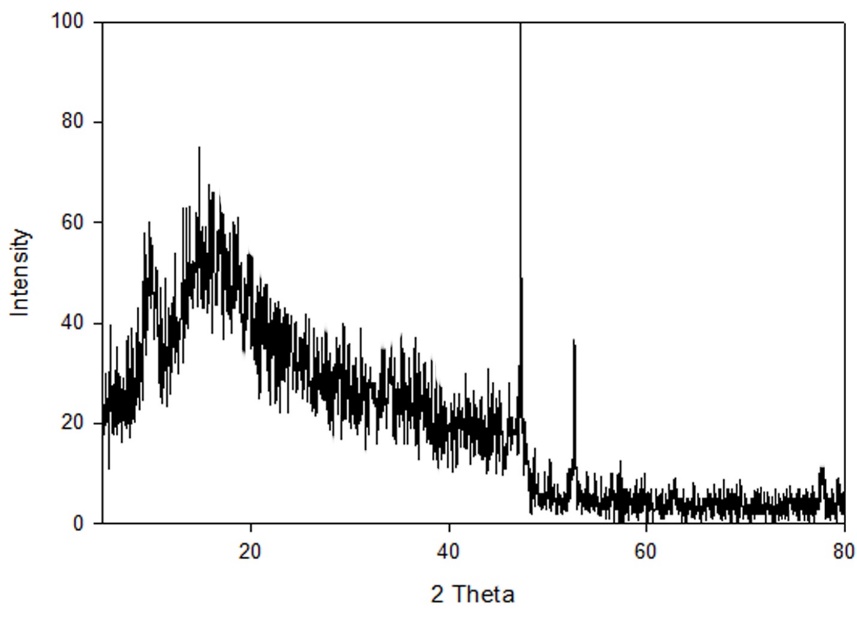
**

**Figure S2.** XRD pattern of NF4


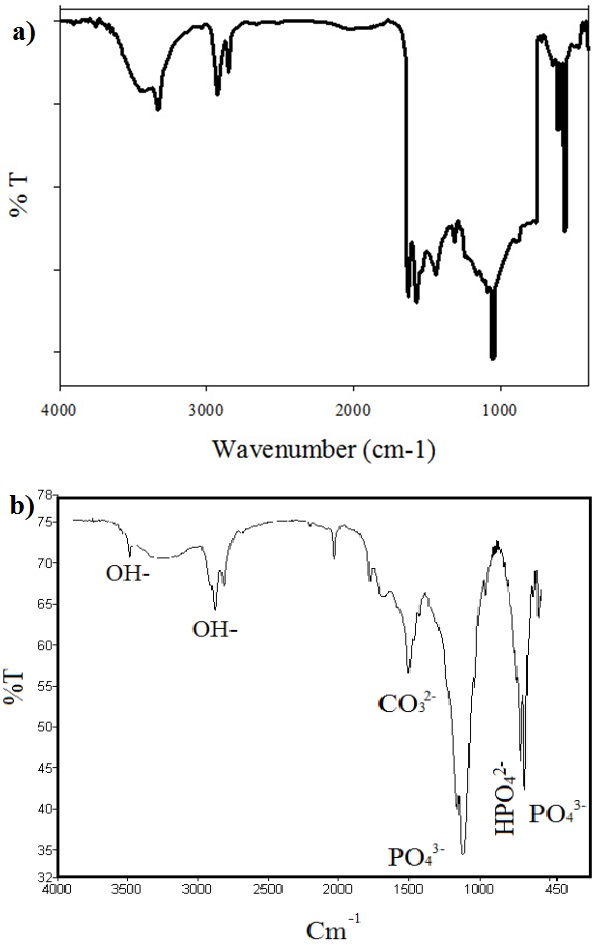


**Figure S3.** The FT-IR spectrum of NF4 in SBF solution after 28 days (a) and the FT-IR spectrum of hydroxyapatite (b) with License Number 5361471042817 from ref. 5.


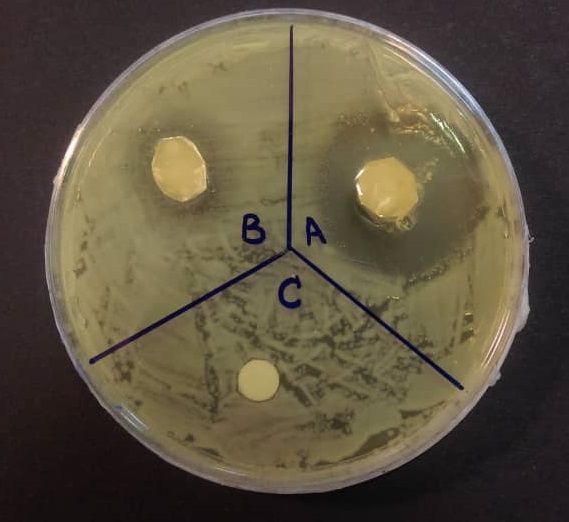


**Figure S4.** Antibacterial activity of A) NF4 sample, B) MTN free NF4 sample, and C) empty disc. Zone of inhibition diameter for A) 2.7 cm, B) 1.3 cm, and C) 0 cm.

| Sample | Fiber diameter (nm) | Porosity (%) | Electrical conductivity (μs) |
| --- | --- | --- | --- |
| NF1 | 445 ± 99 | 65.81 | 8.22 ± 1.22 |
| NF2 | 437 ± 88 | 65.44 | 11.56 ± 1.73 |
| NF3 | 368 ± 99 | 58.76 | 12.13 ± 1.54 |
| NF4 | 372 ± 96 | 64.24 | 15.15 ± 2.42 |

**Table S1.** Fiber diameter, porosity, and electrical conductivity of NF1, NF2, NF3, and NF4

| Sample | Tensile strength (MPa) | Elongation at break (%) | Modulus (MPa) |
| --- | --- | --- | --- |
| NF1 | 6 ± 1 | 42.3 ± 11.5 | 48 |
| NF2 | 7.7 ± 1.2 | 85.3 ± 10 | 55 |
| NF3 | 11.7 ± 3.2 | 92.6 ± 13.5 | 57 |
| NF4 | 6.1 ± 1.5 | 29.6 ± 13.2 | 50 |

**Table S2.** Tensile strength, elongation at break, and modulus of NF1, NF2, NF3, and NF4

| Sample | Water uptake (%) | Contact angle (◦) |
| --- | --- | --- |
| NF1 | 14.18 ± 0.62 | 126 ± 5.2 |
| NF2 | 32.95 ± 0.34 | 92.3 ± 4.1 |
| NF3 | 33.13 ± 0.96 | 85 ± 3 |
| NF4 | 35.62 ± 0.24 | 83.9 ± 3.2 |

**Table S3.** Water uptake and contact angle of NF1, NF2, NF3, and NF4 nanofiber scaffolds

**References**

1. Müller, A., Krickemeyer, E., Bögge, H., Schmidtmann, M. & Peters, F. Organizational forms of matter: an inorganic super fullerene and keplerate based on molybdenum oxide. *Angew. Chem. Int. Ed.* **37**, 3359-3363, DOI: https://doi.org/10.1002/(SICI)1521-3773(19981231)37:24%3C3359::AID-ANIE3359%3E3.0.CO;2-J (1998).

2. Aghdam, R. M., Najarian, S., Shakhesi, S., Khanlari, S., Shaabani, K. & Sharifi, S. Investigating the effect of PGA on physical and mechanical properties of electrospun PCL/PGA blend nanofibers. *J. Appl. Polym. Sci.* **124**, 123-131, DOI: https://doi.org/10.1002/app.35071 (2012).

3. Kokubo, T. & Takadama, H. How useful is SBF in predicting in vivo bone bioactivity? *Biomaterials* **27**, 2907-2915, DOI: https://doi.org/10.1016/j.biomaterials.2006.01.017 (2006).

4. Graham, L. & Orenstein, J. M. Processing tissue and cells for transmission electron microscopy in diagnostic pathology and research. *Nat. Protoc.* **2**, 2439-2450, DOI: https://doi.org/10.1038/nprot.2007.304 (2007).

5. Gheisari, H., Karamian, E. & Abdellahi, M., A novel hydroxyapatite–Hardystonite nanocomposite ceramic. *Ceram. Int.* **41**, 5967-5975, DOI: https://doi.org/10.1016/j.ceramint.2015.01.033 (2015).
